# Analysing black phosphorus transistors using an analytic Schottky barrier MOSFET model

**DOI:** 10.1038/ncomms9948

**Published:** 2015-11-13

**Authors:** Ashish V. Penumatcha, Ramon B. Salazar, Joerg Appenzeller

**Affiliations:** 1 Birck Nanotechnology Center, Purdue University, West Lafayette, Indiana 47907, USA; 2 School of Electrical and Computer Engineering, Purdue University, West Lafayette, Indiana 47907, USA

## Abstract

Owing to the difficulties associated with substitutional doping of low-dimensional nanomaterials, most field-effect transistors built from carbon nanotubes, two-dimensional crystals and other low-dimensional channels are Schottky barrier MOSFETs (metal-oxide-semiconductor field-effect transistors). The transmission through a Schottky barrier-MOSFET is dominated by the gate-dependent transmission through the Schottky barriers at the metal-to-channel interfaces. This makes the use of conventional transistor models highly inappropriate and has lead researchers in the past frequently to extract incorrect intrinsic properties, for example, mobility, for many novel nanomaterials. Here we propose a simple modelling approach to quantitatively describe the transfer characteristics of Schottky barrier-MOSFETs from ultra-thin body materials accurately in the device off-state. In particular, after validating the model through the analysis of a set of ultra-thin silicon field-effect transistor data, we have successfully applied our approach to extract Schottky barrier heights for electrons and holes in black phosphorus devices for a large range of body thicknesses.

## Abstract (web summary)

Conventional models of transistors are not applicable to devices made from nanomaterials because their operation is dominated by gate-dependent transmission through a Schottkybarrier. Here, the authors develop an analytical model and compare it to data taken from ultrathin silicon field-effect transistors.

## Graphical abstract

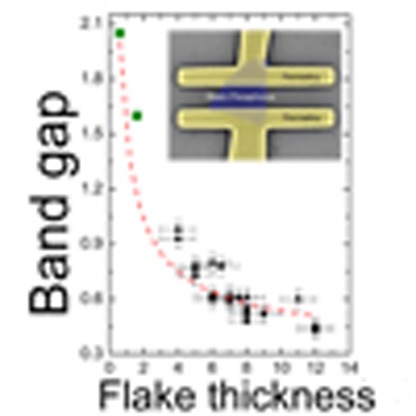

Since the first carbon nanotube transistors were built and characterized in 1998 (refs 1, 2) and found to behave as Schottky barrier metal-oxide-semiconductor field-effect transistors (SB-MOSFETs) in 2002 (refs 3, 4) many more ultra-thin body devices have been explored. Whether in the case of ultra-thin silicon slab structures^5^, silicon nanowires^6^, III–V nanowires^7^ or more recently transition metal dichalcogenides (TMDs)^8^,^9^,^10^, all of these exploratory three-terminal devices with metallic source and drain contacts and a channel that is gated from the source-to-channel to the drain-to-channel interface showed a variety of characteristics that are common for SB-MOSFETs. Different from conventional MOSFETs, SB-MOSFETs show, for example, inverse sub-threshold slopes 
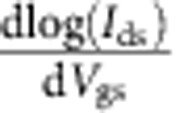
 larger than 60 mV dec^−1^ at room temperature close to the threshold voltage *V*_th_ for finite oxide thicknesses even if the depletion capacitance is zero and interface trap capacitances can be ignored^11^ and so-called ambipolar device characteristics, that is, electron transport for positive gate voltages and hole transport for negative gate voltages^12^. Using conventional MOSFET models to analyse data from SB-MOSFETs often leads to incorrect conclusions about the channel material. In an SB-MOSFET, for example, calculating mobilities using the transconductance (*g*_m_) underestimates the mobility, since *g*_m_ is limited by the gate-voltage-dependent contact resistance of the Schottky barrier. Hence, it is important to first understand the contact properties before extracting intrinsic properties of the channel. The temptation is to correlate linear *I*_ds_−*V*_ds_ in the low-*V*_ds_ region of the output characteristic with the presence of ‘ohmic' contacts. However, linear, rather than exponential, *I*_ds_−*V*_ds_ characteristics are frequently found in ultra-thin body SB-MOSFETs as a consequence of substantial tunnelling through the source and drain Schottky barriers. In the off-state of the device, scattering in the channel is negligible and the shape of the characteristic is dictated mainly by the line-up of the metal Fermi level with the bands of the semiconductor in the channel. As we will describe in this work, the off-state transfer characteristics provide a window into the contact properties of SB-MOSFETs that cannot be easily extracted otherwise.

Because of the wealth of material and interface properties that can be extracted from the electrical characteristics of a SB-MOSFET, we recently discussed in a number of independent publications how identifying the current at threshold, flatband or at the minimum current point of the *I*_ds_−*V*_gs_ characteristics can be used to determine the bandgap (*E*_g_) involved in current transport as well as the actual Schottky barrier heights (
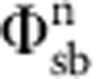
 and 
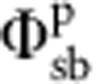
) for electron and hole injection, respectively^9^,^10^. In all of these cases, we have used either the ratio of the current levels at different points in the transfer curve or the actual values of the currents at individual points. One drawback of this technique is the uncertainty associated with identifying these individual points at which the current values need to be compared. Also depending on the exact nature of the *I*_ds_−*V*_gs_ characteristics, the extraction method has to be modified. Ideally, rather than evaluating distinct current levels of the device, the entire off-state device characteristics should be described by our Schottky barrier model. Here we use for the first time a full implementation of the previously only partly discussed analytical SB-MOSFET model for ultra-thin body channel materials, describing the device off-state as a function of both gate and drain voltages. In particular, it is shown that the proposed analytical model can quantitatively explain SB-MOSFET device characteristics with high accuracy for many low-dimensional channel materials without the need to resort to developing new models every time a novel nanomaterial is discovered.

Here we first describe the details of the SB-MOSFET model and validate it by extracting the Schottky barrier heights for a silicon SB-MOSFET. We then apply the model to a newly rediscovered two-dimensional (2D) material—black phosphorus (BP), to extract critical material and interface properties.

## Results

### Schottky barrier field-effect transistor model

A SB-MOSFET consists of a semiconducting channel contacted by metal–source/drain electrodes along with a gate terminal that modulates the potential of the channel. The doped source/drain regions, which form a p–n junction with the channel material, in conventional transistors are replaced with metal contacts. One consequence of this change in the structure of the device is that the n- or p-nature of the MOSFET is dictated by the line-up of the source/drain metal Fermi level to the semiconductor bands rather than the doping type of the channel. One may expect that a line-up of the metal Fermi level close to the valence/conduction band results in a unipolar p/n field-effect transistor (FET). However, the ultra-thin body nature of materials typically used for SB-MOSFETs makes the characteristic length scale *λ* (refs 6, 13) over which band bending occurs at the metal-to-semiconductor interfaces rather small. The *λ*, which defines the shape of bands at the metal–semiconductor interface, for devices with bulk semiconductor channels is controlled by the doping level of the channel. Increasing the doping of the semiconductor reduces the depletion width and allows the electrons to tunnel through the barrier with a higher probability. In an ultra-thin body device, however, the body thickness is much less than the depletion width. Hence, *λ* is now defined by the body thickness, that is, 
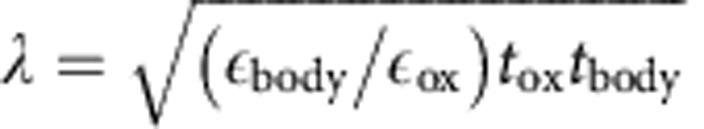
 (Supplementary Note 1). When the semiconductor channel thickness is only a few nanometres, the tunnelling probability through both electron and hole barriers becomes large and this gives rise to ambipolar transfer characteristics. In the ideal limit of *λ*→0, the shape of the transfer characteristic becomes independent of the line-up between the metal Fermi level and the semiconductor bandgap. Evidence for the change in the *λ* dependence can be found in particular by comparing the scaling behaviour of SB devices and conventional devices, and has been discussed by us for TMD FETs in the context of the *t*_body_ impact, that is, TMD flake thickness^14^.

**Figure 1:**
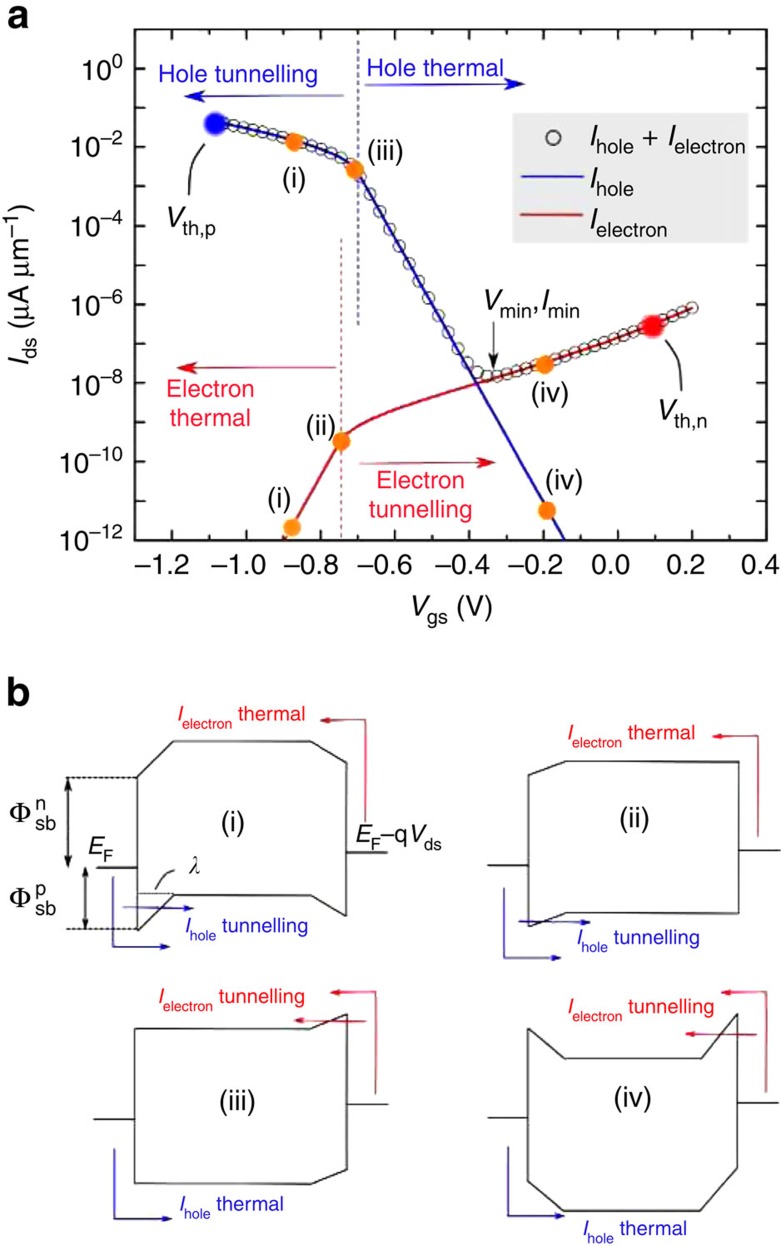
A typical Schottky barrier MOSFET transfer characteristic. (**a**) Plot of the calculated transfer characteristic of an SB-MOSFET for *V*_ds_=−50 mV with 
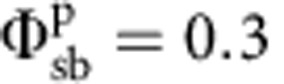
 and 
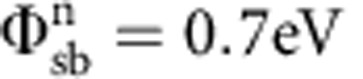
. The total current, *I*_ds_ (open circles) is the sum of *I*_electron_ and *I*_hole_, plotted in red and blue, respectively. Key *V*_gs_ points on the transfer curve are labelled (i)–(iv), with the representative band diagrams drawn in **b**. (**b**) At each *V*_gs_ point labelled (i)–(iv), *I*_hole_ (blue arrows) and *I*_electron_ (red arrows) are each made up of either thermionic emission current (thermal region) or a combination of tunnelling current and thermionic emission current (tunnelling region). (ii) and (iii) are the flat band voltages *V*_fb,D_ and *V*_fb,S_, respectively.

If we assume a metal line-up close to the valence band (
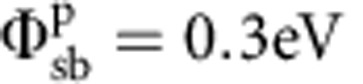
 and 
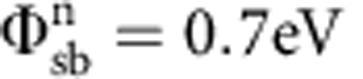
 (Fig. 1b(i)), the p-branch/hole current of the transfer characteristics is higher than the n-branch/electron current. An example of the expected *I*_ds_−*V*_gs_ at room temperature for such a line-up is plotted using open black circles in Fig. 1a. The total current *I*_ds_ can be decomposed into two branches: *I*_hole_ (shown in blue in Fig. 1a) and *I*_electron_ (shown in red in Fig. 1b). Key points in the transfer characteristic have been labelled (i)–(iv) to highlight the different regions in each branch. The *I*_hole_ branch can be broken up into two separate regions, the thermal region and the tunnelling region, separated by a transition point called the flatband voltage. The band diagram at flatband voltage (*V*_fb,S_) for *I*_hole_ is shown in Fig. 1b(iii). As is evident from Fig. 1b(iii), at this gate voltage the bands on the semiconductor side of the metal–source interface side are flat. In the hole thermal region (*V*_gs_>*V*_fb,S_, Fig. 1b(iv)), *I*_hole_ is a pure thermionic emission current over the barrier defined by the valence band in the channel region. As the valence band in the channel is lowered by applying a higher positive *V*_gs_ the barrier becomes larger and the current decreases exponentially. The ideal inverse sub-threshold slope of the *I*_ds_−*V*_gs_ in this region, when the interface trap and depletion capacitances are both zero, is ≈60 mV dec^−1^ at room temperature. In the hole tunnelling region (*V*_gs_<*V*_fb,S_, Fig. 1b(i)), *I*_hole_ is a combination of thermionic emission and tunnelling currents. The barrier for thermal emission of holes is now fixed at 
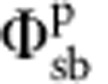
 and the thermionic component no longer increases exponentially with decreasing gate voltage. The tunnelling component on the other hand increases because the tunnelling barrier becomes thinner as the gate voltage is made more negative. The sum of these two components exhibits an inverse sub-threshold slope defined by the tunnelling probability^6^.

All of these features of an SB-MOSFET can be accurately captured in a simple Landauer picture^15^,^16^ because scattering is not significant in the off-state. Instead, the transmission *T*(*E*) through the device is dominated by the Schottky barriers at the source and drain (Supplementary Note 2 and Supplementary Fig. 1). Also, the channel potential can be modulated linearly with gate voltage if we assume that the interface trap capacitance is approximately constant across the bandgap. The current per unit width *I*_ds_ through the device can be written as


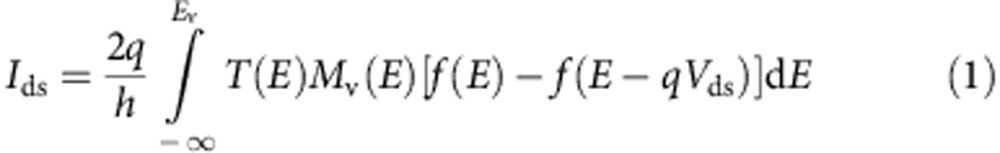


where 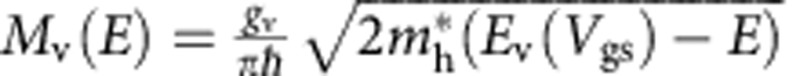, where *E*_v_>*E* represents the number of modes per unit width in the 2D channel, *g*_v_ is the valley degeneracy and 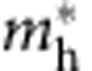 is the hole effective mass. *T*(*E*) is the net transmission through the source and drain Schottky barriers and *f* is the Fermi–Dirac function. *V*_ds_ is the drain-source voltage that drives current flow in the device and *E*_v_ is the valence band edge in the gate-controlled channel region (the part of the channel that is controlled only by the gate terminal) of the SB-MOSFET. In our model, we assumed the barriers to be triangular in shape, with a base width of *λ* (Fig. 1b) at any gate voltage. We then used a semi-classical WKB approximation to calculate the tunnelling probability (*T*_s_ and *T*_d_) of the source and drain junctions separately. The transmission *T*(*E*) through the device was calculated as 
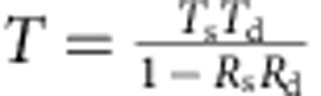
 (refs 15, 16) where *R*_s(d)_=1−*T*_s(d)_. The gate voltage moves the flat portion of the channel region up/down and the shape of the barrier at the source and drain changes accordingly (Fig. 1b). Thus, the applied gate voltage determines the channel potential, which changes the available modes *M*(*E*_v_−*E*) in the channel and also the transmission through the source and drain tunnelling barriers.

For a triangular-shaped barrier, the probability of tunnelling through the forbidden region into the valence band is calculated using the semi-classical one-dimensional WKB approximation as:


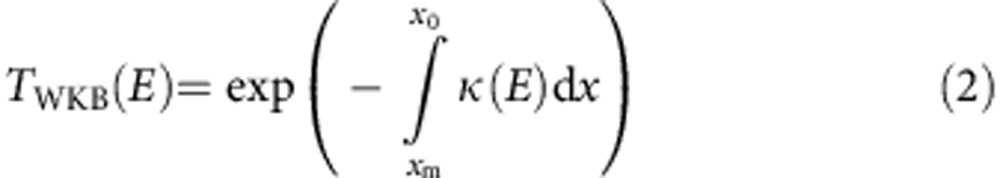



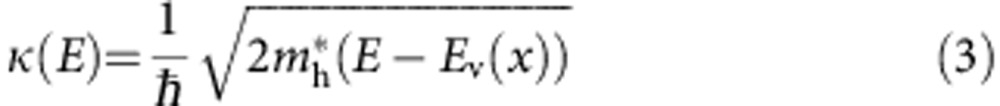


where *x*_m_ (at the metal–semiconductor (M–S) junction) and *x*_0_ (*x* at which *E*=*E*_v_) are the positions of two classical turning points at energy *E*, *E*_v_(*x*) is the position-dependent valence band energy maximum, 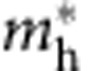 is the effective mass of the valence band and *E* is the energy of interest. At each gate voltage, the energy integral in equation (1) was evaluated to calculate the current in the valence band. The *I*_ds_−*V*_gs_ for the hole branch at a fixed negative *V*_ds_ is plotted using a blue line in Fig. 1a.

For a negative *V*_ds_ voltage, electrons flowing from the drain to the source through the conduction band also contribute to the total current (as shown in Fig. 1b with red arrows). The electron contributions to the total current can be analysed in an identical fashion to the hole current described above. The red line in Fig. 1a shows how *I*_electron_ varies with gate voltage. As discussed for *I*_hole_, we can divide *I*_electron_ into two regions. The region between (i) and (ii) corresponds to the thermal branch and between (ii) and (iv) corresponds to the tunnelling branch. (ii) is the flatband voltage (*V*_fb,D_) for the electron branch. Because of the assumptions we have made about the voltage drop being negligible in the channel, we can write *V*_fb,D_−*V*_fb,S_≈*V*_ds_. The region between (i) and (ii) exhibits a 60 mV dec^−1^ inverse sub-threshold slope at room temperature. Similar to the hole branch the slope in the region between flatband and (iv) is proportional to the tunnelling probability. Note in particular that in the example shown in Fig. 1, the hole current is independent of *V*_ds_, while the electron current varies exponentially with drain voltage.

In summary, for the 
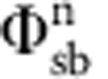
 and 
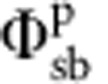
 values chosen here, in the total current *I*_ds_ one can identify the thermal branch of *I*_hole_ and the tunnelling branches of *I*_electron_ and *I*_hole_. The entire *I*_ds_−*V*_gs_ curve can be described by the Schottky barrier heights 
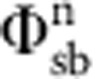
 and 
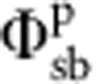
 for a given geometric screening length (*λ*), *V*_ds_ and effective masses in the conduction and valence bands. The current level on the far p-side (region between (i) and (iii)) is proportional to 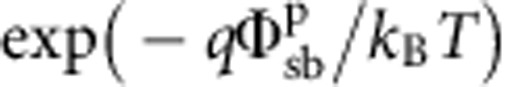. At the minimum current point, the current for an asymmetric line-up described above is the sum of a thermionic emission current through the valence band and the combination of thermionic and tunnelling current through the conduction band. The current level to the right of the minimum point is approximately proportional to 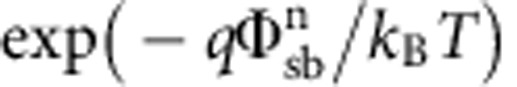. In addition to the barrier height 
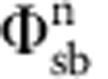
, the current level is also impacted by the *V*_ds_ value. The slope of the section of the transfer curve to the right of the minimum point (Fig. 1a) is a function of the tunnelling probability through the drain barrier.

Given a measured *I*_ds_−*V*_gs_ data set of an SB-MOSFET, we can fit to the data using 
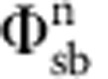
 and 
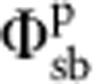
 as input parameters to the SB-MOSFET model. Other input parameters to the model include the geometric screening length *λ*, the effective mass and the applied *V*_ds_—all of which are typically known experimental/theoretical parameters. The fit allows us to estimate the individual Schottky barrier heights and the bandgap (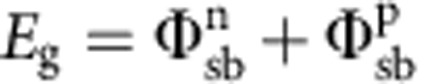) from the off-state *I*_ds_−*V*_gs_ data, that is, any current data between the threshold voltages of the device.

The threshold voltages (*V*_th,n_ and *V*_th,p_) are the gate voltages at which the tunnelling through the 
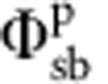
 for holes and 
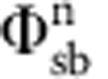
 for electrons makes the barriers almost transparent. Beyond threshold, the number of carriers injected into the channel becomes large enough that to simulate the device behaviour, one needs to solve the Poisson's equation self-consistently with an accurate transport model, which includes scattering in the channel. The off-state of the device is the gate voltage range between *V*_th,n_ and *V*_th,p_ where transmission through the barriers is small. In this gate voltage range, one can neglect scattering in the channel without making a significant error in the overall estimated current value (Supplementary Note 2 and Supplementary Fig. 1).

For an ultra-thin body device biased in the off-state, the semiconductor capacitance *C*_Q_≈0 and *C*_D_≈0. The body factor can be written as *γ*=1+*C*_it_/*C*_ox_. The presence of the interface trap capacitance makes the sub-threshold slope in a real device deviate from its ideal slope. To fit the experimental data, we assume that *C*_it_ is constant as mentioned before and simply rescale the *V*_gs_ axis by *γ*. The curves are also offset along the *x* axis to account for *V*_th_ shifts from device to device, which are a result of unintentional doping, surface adsorption of charged species and hysteresis in the measured transfer characteristics. All these effects only result in a rigid *x* shift of the transfer characteristic of a ultra-thin body device. Using this approach, the following sections focus on the validation of our model and the extraction of relevant material parameters. The rescaling of the *V*_gs_ axis is the only additional fitting parameter besides 
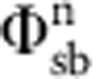
 and 
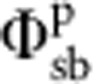
, and does not impact the extraction of 
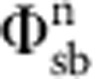
 and 
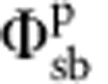
.

### Validating the model

**Figure 2:**
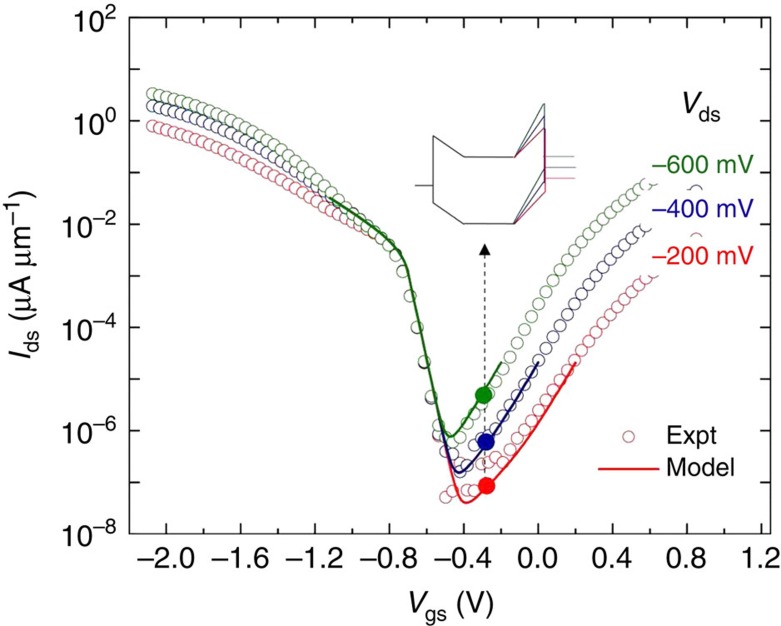
Validating the model. Transfer characteristic of a silicon SB-MOSFET with *L*_ch_=2 μm, *t*_ox_=3 nm and *t*_Si_=25 nm from Knoch *et al.*^5^ Experimental data are plotted in red, blue and green open circles. The fits to the different *V*_ds_ curves using our SB-MOSFET model are plotted using solid lines of the corresponding colour. The same 
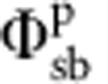
 and 
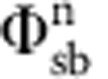
 where used to fit all three *I*_ds_−*V*_gs_ curves for different *V*_ds_ values. The inset shows the band bending situation for different *V*_ds_-voltages at a fixed *V*_gs_.

Because silicon is one of the most well-understood semiconductors, in this section we have used the model described above to first fit the measured *I*_ds_−*V*_gs_ of an ultra-thin body silicon SB-MOSFET from Knoch *et al.*^5^ The geometric screening length was calculated from the device dimensions to be ∼15 nm. The effective mass for electrons and holes was taken to be 0.2 and 0.35 (ref. 17; Supplementary Note 3). For 
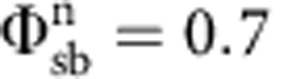
 and 
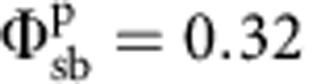
, we observe excellent agreement between our SB-MOSFET model and the experimental data set (Fig. 2) over the entire off-state. Note that the value of 
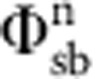
 is slightly larger than quoted by Knoch *et al.*^5^ The validity of the SB-MOSFET model is further demonstrated by fitting to experimental data for different *V*_ds_. Note that the same parameters were used to fit transfer curves for all three *V*_ds_ values. The calculated curve was truncated at a gate voltage point where we believe that barriers become transparent enough that one can no longer justify the off-state assumption. As is evident from Fig. 2, increasing *V*_ds_ to more negative values does not change the hole current (before the threshold voltage is reached) but the electron current increases exponentially.

In an electrostatically well-behaved device, applying a certain *V*_ds_ does not change the shape or height of the barrier at the source. Therefore, we do not expect to observe an exponential change in the *I*_ds_. However, for SB-MOSFETs, the shape of the barrier on the drain side is impacted by the drain voltage. In our example in Fig. 1, since *I*_electron_ is the tunnelling current through the drain-side electron Schottky barrier, it is expected to increase exponentially if the tunnelling probability through this barrier increases. If we assume perfect gate control of the channel potential, *V*_ds_ drops entirely across the drain junction. This implies that our model cannot predict any DIBL, since ideal electrostatics are assumed. The more negative the applied *V*_ds_ the thinner the barrier on the drain side becomes (inset in Fig. 2). Alternatively, one could say that, for negative *V*_ds_ values, *V*_fb,D_ occurs earlier, at more negative *V*_gs_ values. This means that the current at point (iv) in Fig. 1a is expected to increase with *V*_ds_.

In summary, the off-state characteristics of an ultra-thin body silicon SB-MOSFET are successfully described, without any fitting parameters, for a wide range of gate and drain voltages within a straight forward analytical model. It is quite interesting that a model, which treats the SB-MOSFET as two ‘gateable' Schottky barrier diodes connected back to back, neglecting all inelastic scattering events in the channel, can accurately describe the off-state current through the device. However, as evidenced by the quality of the fits to the experimental data, our SB-MOSFET model can not only describe qualitatively the shape of the transfer characteristic, it can also quantitatively describe the *I*_ds_−*V*_gs_ in the off-state along with the *V*_ds_ dependence for small voltages. With this framework in place, we are in a position to evaluate the electrical characteristics of newer low-dimensional materials.

### Black phosphorus SB-MOSFETs

BP is a newly rediscovered 2D material with several unique properties. It is a layered material with each layer consisting of phosphorus atoms arranged in a puckered structure with strong in-plane anisotropic properties that have sparked several ideas for thermoelectric^18^,^19^, optoelectronic^20^,^21^ and spintronic devices^22^. Its high mobility and moderate bandgap (in the bulk form) also makes it attractive for radio frequency applications^23^,^24^,^25^. Several density functional theory (DFT) calculations have pointed out the fact that the bandgap is a strong function of layer number but the predicted bandgap varies depending on the exchange-correlation energy functional used for DFT calculations. In this section, we have used our SB-MOSFET model to extract bandgap and Schottky barrier information from measured data of BP SB-MOSFETs of different body thicknesses.

BP thin films were isolated from a bulk piece of single-crystal BP purchased from Smart Elements (purity 99.998%) using mechanical exfoliation and transferred onto a 20-nm thermally grown SiO_2_ on highly doped silicon. Standard electron beam lithography was used to pattern permalloy (Ni_81_Fe_19_) source-drain contacts on BP thin films of different thicknesses. Care was taken to ensure that the substrate with the BP flakes was immediately coated with resist and processed within a few hours and loaded into a vacuum chamber to prevent any degradation of the BP flakes^26^,^27^. The electrical measurements of the devices were carried out in a vacuum chamber at a pressure of ≈5e-5 Torr. Using the same approach, some SB-MOSFET devices were also made with palladium contacts for comparison. After the electrical measurements, the flake thickness of the devices was measured using an atomic force microscope (AFM) operated in the tapping mode. When the height of a flake is measured with respect to the substrate using tapping-mode AFM, it has been reported^28^ that thickness variations of the order of 0.5–1 nm are common. Also, since the AFM scan was recorded after the entire fabrication and measurement, additional adsorbates may be present on the surface of the BP flake. Taking into account these uncertainties in the thickness measurements, a generous error bar of 1 nm is assumed.

**Figure 3:**
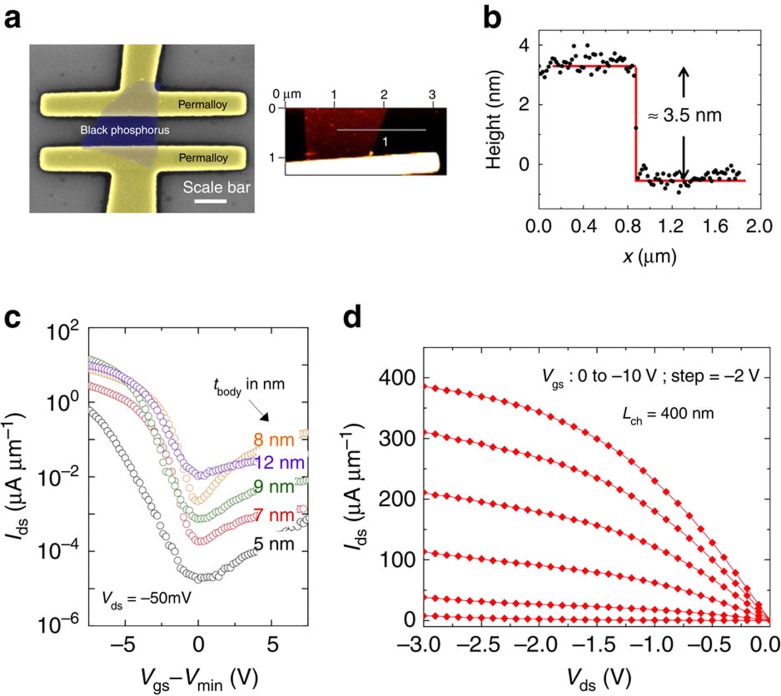
*t*_body_ dependence of measured electrical data from BP SB-MOSFET. (**a**) False-coloured SEM image of a BP SB-MOSFET with permalloy contacts. The scale bar represents a length of 400 nm. All devices were fabricated in a back-gated geometry with thermally grown 20-nm-thick SiO_2_ as the gate oxide. (**b**) Representative AFM image of a BP flake and a line scan used to determine the flake thickness (**c**) Transfer characteristics at *V*_ds_=−50 mV for SB-MOSFETs with permalloy contacts on BP for different flake thicknesses (*t*_body_). Note that device-to-device variations occasionally result in ‘outlier' devices, which are included in our analysis (Fig. 4b,c). (**d**) Room temperature output characteristic of a 8-nm thick BP SB-MOSFET with permalloy contacts.

Figure 3d shows representative output characteristics of one of our BP devices. The high hole on-current (maximum measured current) in the range of several hundreds of μA μm^−1^ suggests that the permalloy Fermi level lines up close to the valence band edge of BP. The actual values of the on-currents are expected to be a strong function of layer thickness due to an interplay of scattering from the substrate and the interlayer resistance^9^. Note that the apparent linear *I*_ds_−*V*_ds_ for small *V*_ds_ is not evidence of the absence of Schottky barriers. The sub-threshold slopes and the threshold voltages are subject to device-to-device variations. The source of these variations is still an active area of research for carbon nanotubes and other low-dimensional channel materials^29^,^30^. For this reason, the curves in Fig. 3c are offset along the *x* axis to allow for a comparison of device characteristics for different *t*_body_. From Fig. 3c, it is apparent that there is a trend in the minimum current for different body thicknesses, that is, *I*_min_ (Fig. 2) is increasing with increasing body thickness. As will be discussed below, this trend is mainly a result of the changing bandgap and Schottky barrier heights as a function of BP body thickness.

Next, we use our SB-MOSFET model described above to extract the Schottky barrier heights 
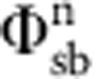
 and 
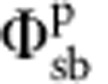
 for the permalloy- and palladium-contacted BP SB-MOSFETs. The Schottky barrier height extracted from the permalloy devices for different layer thicknesses can guide more detailed experiments that probe spin injection and transport in BP. In addition, the sum of 
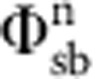
 and 
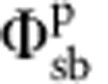
 allows us to determine the bandgap of BP at different body thicknesses.

**Figure 4:**
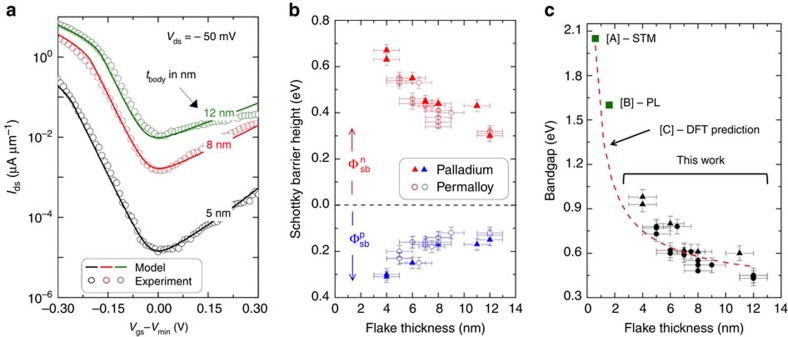
Using the SB-MOSFET model to extract SB heights of BP. (**a**) Plots showing fits to the experimental data (open circles) using our SB-MOSFET model. The *x* axis of the experimental *I*_ds_−*V*_gs_ data was rescaled to account for the effect of *C*_it_ as discussed in the text. (**b**) The extracted Schottky barriers for different flake thicknesses of BP. Open circles (blue and red) represent SB-MOSFETs with permalloy contacts. Filled triangles (blue and red) represent SB-MOSFETs with palladium contacts. The *y* error bar captures the uncertainty in the measured transfer characteristic of a single device due to commonly observed hysteresis effects, charging of the substrate and so on. It also captures the uncertainty associated with the fit. The *x* error bar captures the error in the AFM thickness measurement (see text). (**c**) Plot of bandgap versus flake thickness. *E*_g_ was calculated as the sum of the electron and hole Schottky barriers from **b**. Our data are in excellent agreement with a power-law fit from DFT simulations reported by Tran *et al.*^37^ Green squares [A]^39^ and [B]^40^ are experimental data from the literature.

For the analysis of the permalloy- and palladium-contacted BP SB-MOSFET data, the voltage axis of the transfer characteristics of the BP SB-MOSFETs was rescaled by a constant factor as described for the silicon SB-MOSFET case. The data were then fit using the model to extract 
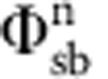
 and 
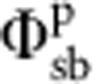
 separately for permalloy and palladium contacts. The fits for a few selected flake thicknesses are shown in Fig. 4a. The only other input parameters to the model were the effective masses 
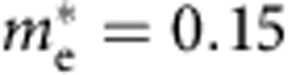
 and 
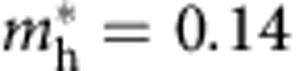
 from literature^31^. 
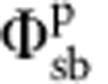
 and 
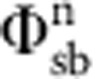
 were the only two free parameters used to fit to the experimental data. For this simulation the characteristic length scale *λ* was assumed to be 
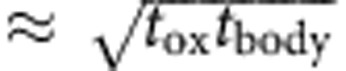
 (ref. 10). The extracted barrier heights for permalloy (blue and red open circles) and palladium (blue and red filled triangles) are plotted in Fig. 4b. Similar to other reports^32^,^33^,^34^ in literature for metal contacts to BP, we find that the Fermi level of the metal is pinned close to the valence band of BP irrespective of whether palladium or permalloy is used as the contact metal. Our results show in particular that in terms of Schottky barrier height permalloy behaves rather similar to palladium, a large work function metal.

Using our model we extract, in particular, a barrier height of ≈120 meV for a 12-nm-thick flake with permalloy contacts. This value is in agreement with the 110 meV barrier height measured by Anugrah *et al.*^35^ using the standard Arrhenius technique^9^,^36^. Because our SB-MOSFET model allows easy access to Schottky barrier heights, we are able to analyse how the barrier height changes for the different body thicknesses without having to perform low-temperature measurements for each device. It is also worth noting that the standard Arrhenius technique can be used to measure only one of the Schottky barrier heights, while our SB-MOSFET model provides both the electron and hole Schottky barrier heights from a single data set.

The reader might wonder at this point why the above analysis did not take into account the anisotropic transport conditions in BP since it is a well-known fact that the band structure of BP is a strong function of layer thickness and crystallographic direction^31^. In particular, the effective mass in the zigzag direction is heavier by a factor of 6–8 when compared with the armchair direction. Moreover, the band structure also changes as a function of layer thickness. Please note in this context that all the data analysed in this study were obtained from BP flakes thicker than 4 nm and that DFT simulations^31^,^37^ show that the effective masses of both electrons and holes do not change significantly as the number of layers increases beyond 2–3. Regarding the anisotropic transport in BP, our wide channels are key to understanding the use of low effective masses for electrons and holes. For a wide device, charged carriers impinging on the metal-to-semiconductor interface with a random distribution of momenta will be preferably transmitted for small effective masses since the current will flow along the least resistive path from the contacts into the channel. Thus, it is expected that the lower of the two (armchair and zigzag) effective masses dominates the tunnelling process. Moreover, the Schottky barrier height extracted from our model is rather robust to uncertainty in the effective mass value as discussed in the Supplementary Note 4 and Supplementary Fig. 2. For a device with the same contact metal at the drain and the source, we can sum 
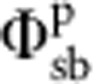
 and 
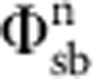
 in Fig. 4b to estimate the bandgap for each flake thickness. The extracted bandgap versus flake thickness (filled circles and triangles) is plotted in Fig. 4c. The bandgap decreases with increasing flake thickness, which is in qualitative agreement with DFT calculations in literature^31^.

## Discussion

First reports of DFT calculations of monolayer BP within the PBE, HSE functionals predicted a bandgap (≈0.9–1.5 eV for monolayer BP)^31^,^38^. On the other hand, for 4-nm-thick flakes we extract a bandgap of *E*_g_ ≈0.95 eV, suggesting that the monolayer bandgap should be much higher than the aforementioned DFT values. Tran *et al.*^37^ showed that the GW-DFT calculations results in a bandgap of around 2 eV for monolayer BP. The rather large self-energy correction, which led Tran *et al.* to conclude that the bandgap is higher than the first DFT calculations, is expected to be especially important for BP due to the quasi-one-dimensional nature of the bands, which is a result of the strong anisotropy in the BP band structure^32^,^33^. The red dashed line (Fig. 4c) is a power-law fit of the bandgap versus flake thickness from Tran's DFT calculations. The bandgap for a monolayer BP was also experimentally determined ([A] in Fig. 4c) by Liang *et al.*^39^ using a scanning tunnelling microscope. Moreover, photoluminescence measurements^40^ performed on 1.6-nm-thick BP flakes showed a peak of ≈1.6 eV ([B] in Fig. 4c). In general, photoluminescence measurements measure an optical gap that is expected to be lower than the bandgap by the exciton binding energy if we assume that the material is intrinsic and impurity levels do not interfere with the photoluminescence measurement. This implies that the data point [B] underestimates the transport gap in an intrinsic BP flake, in line with the expectation that the true bandgap of thin BP flakes is larger than initially predicted by earlier DFT calculations.

Note that in both of these reports^39^,^40^ the exfoliation and transfer of BP flakes was performed in an inert environment. Figure 3c also contains the ‘outlier' data mentioned before. For example, one can see that the minimum current for this 8-nm device is higher than for the 9-nm device. Figure 4b,c also reveal that the spread in the extracted 
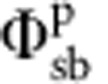
, 
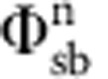
 and *E*_g_ at a particular flake thickness is larger than the individual *y* error bar for each data point. The accuracy of the extracted value of the Schottky barriers and the bandgap at a given flake thickness is limited by the device-to-device variations in the fabricated SB-MOSFETs rather than the model itself.

The extracted bandgaps presented in this work span a flake thickness range of 4–12 nm and are a strong validation of the power-law fit from DFT calculations by Tran *et al.*^37^ However for the same layer thicknesses, Das *et al.*^34^ reported smaller bandgaps (≈0.55 eV for a 4-nm-thick flake) than those predicted by our work as well as other experimental results ([A] and [B]) plotted in Fig. 4c. Likely, our more complete approach that describes the transfer curve over the entire off-state rather than exploiting individual current levels is responsible for the more accurate data analysis presented here.

In all of the above device characteristics, barriers larger than 3–4 *k*_B_*T* and *λ* values above 10 nm determined the injection properties. When analysing transfer characteristics of a SB-MOSFET where the Schottky barriers are transparent, the kink that we observe at *V*_fb_ when transitioning from the thermal to the tunnelling branch becomes less apparent, making it challenging to interpret the data unambiguously.

**Figure 5:**
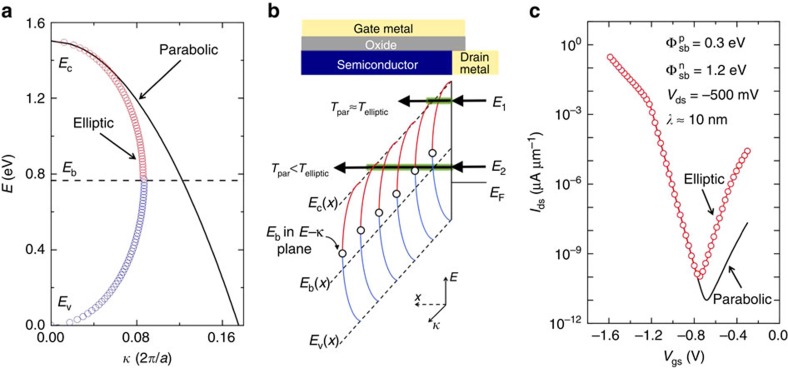
Impact of elliptic *κ*(*E*) on the tunnelling current through a Schottky barrier. (**a**) Complex band structure *κ*(*E*) for *E*_v_<*E*<*E*_c_. The parabolic approximation is valid only close to the band edges. The elliptic *κ*(*E*) is parabolic near the band edges with the effective mass of the corresponding band. Near the branching point 
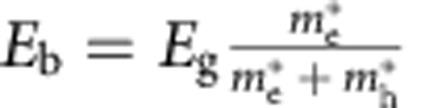
, the complex band becomes non-parabolic. (**b**) Band bending situation for a triangular barrier at a metal–semiconductor junction. The blue and red lines are stitched together according to the elliptic approximation in **a**. The open circle depicts the position of the branching point in the *E*−*κ* plane. The position of *E*_b_ in the *E*−*x* plane is shown using a dashed line. (**c**) Simulated *I*_ds_−*V*_gs_ curve for elliptic and parabolic *κ*(*E*) for specific metal line-up. For tall Schottky barriers, the more commonly employed parabolic *κ*(*E*) can severely underestimate the tunnelling current.

Finally, we would like to provide the reader with an important modification of the above model that becomes relevant for the extraction of Schottky barriers for tall, asymmetric Fermi-level line-ups. The fact that thin layers of BP are susceptible to degradation in ambient atmosphere^26^,^27^ has made electrical characterization of mono-,bi- and other ultra-thin layers extremely challenging. For these flake thicknesses, the Schottky barrier height is expected to increase if the trends in Fig. 4c can be extrapolated to thin flakes. We believe that the tunnelling probability through these tall barriers is likely underestimated by the WKB approximation, which makes use of a single effective mass through the whole bandgap. To model the drain current in an ultra-thin body Schottky barrier FET using the Landauer approach, until now we have employed the semi-classical WKB approximation for a triangular barrier. The mass used to calculate the tunnelling probability is just the effective mass of the electron(hole) for tunnelling into the conduction(valence) band (equation (3)). *κ*(*E*) (*κ* is the imaginary part of the wave vector *k*) for a parabolic band for an energy *E* in the bandgap is plotted in Fig. 5a (black line) for a semiconductor with *E*_g_=1.5 eV and 
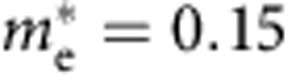
 and 
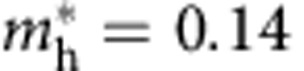
.

For an accurate description of tunnelling through the forbidden region, one needs to consider the complex band structure, which in general involves both conduction and valence branches in the complex plane^41^,^42^,^43^,^44^,^45^. This is routinely done in band-to-band tunnelling and leads to a ‘reduced effective mass' under the assumption that both conduction and valence bands are parabolic. However, close to the branching point (*E*_b_) the bands are elliptic in shape and this can lead to a further increase in the tunnelling probability. Guan *et al.*^43^ observed that the elliptic nature of the complex band structure can be analytically approximated for common semiconductors, especially for direct semiconductors, in a straightforward manner.

This analytical approximation of *κ*(*E*) from Guan *et al.*^43^ is plotted (red and blue) in Fig. 5a. The implicit assumption in using the elliptic *κ*(*E*) is that the complex band structure of the semiconductor is not impacted by the presence of the metal at the interface. Also, we are dealing with a direct bandgap semiconductor where the conservation of the real part of *k* is already satisfied. Close to the conduction(valence) band edge, the parabolic *κ*(*E*) with appropriate effective mass is an accurate description of the complex bands. Therefore, for energies close to the band edges (like the cases considered so far) the tunnelling probability can be captured accurately using the parabolic approximation. Close to the branching point *E*_b_, however, the non-parabolicity of the bands becomes important and the tunnelling probability is underestimated by the parabolic *κ*(*E*) because the action integral overestimates the area bounded by *κ*(*E*) and the energy axis if the parabolic approximation is used (Fig. 5a). In the case of a triangular barrier at the metal–semiconductor junction (Fig. 5b), for energy *E*_1_, where the tunnelling trajectory is close to the conduction band edge, the tunnelling probabilities calculated from the elliptic and parabolic *κ*(*E*) are identical. However, for *E*_2_, the electron path is close to the branching point (*E*_b_(*x*)) for a significant portion of the tunnelling process. This is approximately the scenario for thin BP flakes where the tunnelling barrier on the n-side is larger than 1 eV. Using the elliptical *κ*(*E*) in our SB-MOSFET model, we calculate the *I*_ds_−*V*_gs_ at *V*_ds_=−0.5 V for a bandgap of 1.5 eV, 
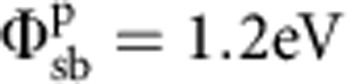
, 
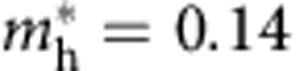
 and 
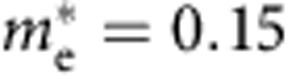
. The comparison between the parabolic and elliptical *κ*(*E*) is plotted in Fig. 5c. As expected, the tunnelling branch for the holes is not impacted since 
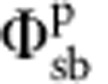
 is small. The biggest change is seen in the tunnelling branch of the electron current. In other words, if one would analyse the minimum current level *I*_min_ using a simple parabolic *κ*(*E*) WKB approximation, one would extract a much larger bandgap since *I*_min_ is severely underestimated. To the best of our knowledge, this is the first time that the non-parabolicity of the complex bands has been applied to Schottky barriers and discussed in the context of device applications.

In conclusion, a novel general analytic model that quantitatively describes the off-state transfer characteristics of SB-MOSFETs using Landauer's formalism has been proposed, validated and used to extract critical properties of multilayer BP devices. Through a detailed comparison of experimental gate- and drain voltage-dependent data of an ultra-thin body silicon FET with the model, it is shown that with the Schottky barrier heights for electron and hole injection as the only free parameters excellent agreement between our analytical description and the measurement can be achieved. Moreover, applying our approach to extract the Schottky barrier heights for multilayer BP devices and evaluating the corresponding dependence of bandgap energy on flake thickness confirmed predictions from DFT calculations, which include self-energy corrections as a critical distinguishing part. Last, as an extension of the simple model, we also proposed the use of elliptic complex bands for calculating tunnelling currents through tall Schottky barriers, an aspect that to the best of our knowledge has not been considered previously. On the basis of our detailed analysis we expect that the analytical model presented here is applicable to a wide range of 2D materials making it a useful tool to gather critical insights into the properties of novel nanomaterials and a platform for future compact modelling efforts.

## Additional information

**How to cite this article**: Penumatcha, A. V. *et al.* Analysing black phosphorus transistors using an analytic Schottky barrier MOSFET mode. *Nat. Commun.* 6:8948 doi: 10.1038/ncomms9948 (2015).

## Supplementary Material

Supplementary Information Supplementary Figures 1-2, Supplementary Notes 1-4 and Supplementary References
