# Corrigendum: Analysing black phosphorus transistors using an analytic Schottky barrier MOSFET model

**DOI:** 10.1038/ncomms11913

**Published:** 2016-06-06

**Authors:** Ashish V. Penumatcha, Ramon B. Salazar, Joerg Appenzeller

Nature Communications 6: Article number: 894810.1038/ncomms9948 (2015); Published: 11132015; Updated: 06062015.

This Article contains an error in the penultimate sentence of the penultimate paragraph. This sentence should read: ‘In other words, if one would analyse the minimum current level *I*_min_ using a simple parabolic *κ*(*E*) WKB approximation, one would extract a much smaller bandgap since *I*_min_ is severely underestimated.'

