# Supplementary material for: Analysing black phosphorus transistors using an analytic Schottky barrier MOSFET model
Source: Nat Commun. 2015 Nov 13;6:8948. doi: 10.1038/ncomms9948 (PMC4660372; doi:10.1038/ncomms9948)
Supplement: Supplementary Information — Supplementary Figures 1-2, Supplementary Notes 1-4 and Supplementary References [file ncomms9948-s1.pdf]

Supplementary Figures

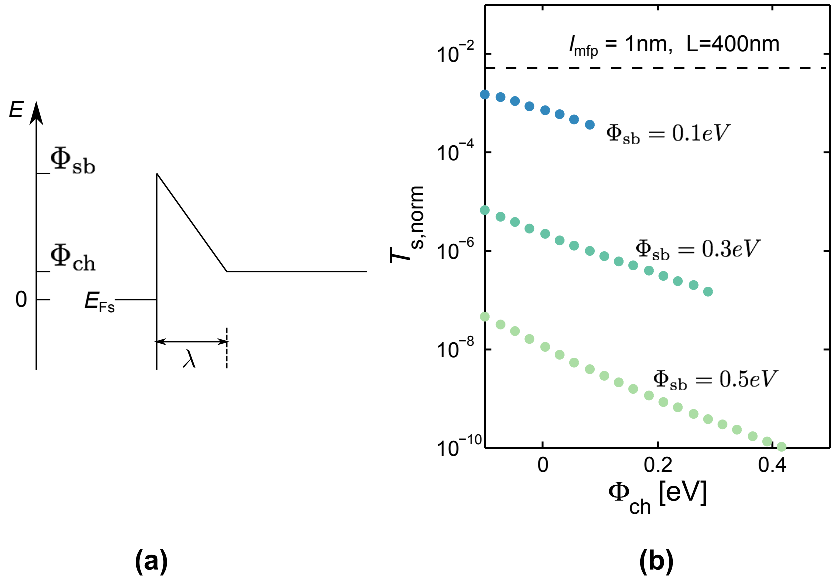

Supplementary fig.1**:** (a) definitions of the channel potential (Φ_{ch}), Schottky barrier height (Φ_{sb}) and energy axis for supplementary note 2. (b) The normalized transmission through the source and drain Schottky barriers ($T_{s,norm}$) for different Schottky barrier heights ranging from 0.1eV to 0.5eV is plotted as a function of channel potential. Even when a very small mean free path is assumed in the channel the transmission through the triangular Schottky barriers still limits the total current.

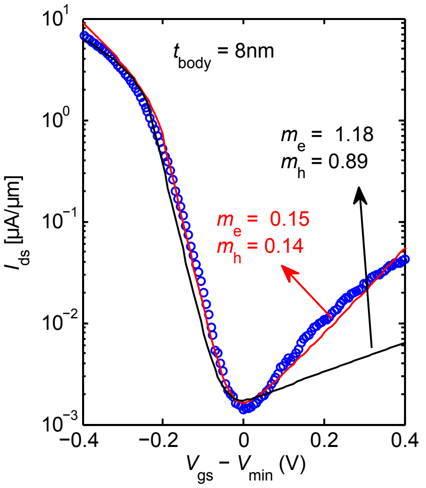

Supplementary fig.2**:** Fit to the measured transfer curve (open blue circles) from an 8nm thick BP flake using two different effective mass pairs corresponding to the armchair (red line) and zigzag (black line) directions in BP.

Supplementary Notes

Supplementary Note 1:
The expression for the length scale λ is in particular valid for our devices because we are analyzing a single-gated structure with SiO_2 as dielectric and the channel length is much larger than λ. For aggressively scaled MOSFETs having multiple gates or high-k dielectrics one should modify the expression for λ or resort to a numerical solution to the Poisson's equation to determine the shape of the bands in the off-state. (Supplementary reference [1])

Supplementary Note 2: When can scattering in the channel be neglected in comparison to the Schottky barrier contacts?

In the paper, we stated that in the off-state the Schottky barriers limit the transmission through the device and that scattering inside the channel can be ignored in comparison. This statement is however not universally true. In this section, we will provide a quick guide to evaluate the applicability of our model for usage in the context of other devices, explicitly considering the impact of channel length.

The total transmission (T) through the device is:

$$T = \frac{T_\mathrm{s} T_\mathrm{d}}{1-\left(1-T_\mathrm{s}\right)\left(1-T_\mathrm{d}\right)}$$

when uncorrelated transmissions T_s and T_d are considered and electron interference phenomena are excluded AND if we assume that the transmission through the channel can be neglected in the off-state since Schottky barriers are limiting T. Including the channel transmission (T_ch) in the expression for T on the other hand, we can write (Supplementary reference 2)

$$T^{-1} = \left(\frac{1-T_\mathrm{s}}{T_\mathrm{s}}\right) + \left(\frac{1-T_\mathrm{d}}{T_\mathrm{d}}\right) + \left(\frac{1-T_\mathrm{ch}}{T_\mathrm{ch}}\right) + 1$$

To neglect T_ch in the above expression,

$$\left(\frac{1-T_s}{T_s}\right) + \left(\frac{1-T_d}{T_d}\right) \gg \left(\frac{1-T_{ch}}{T_{ch}}\right).$$

For small V_ds, $T_\mathrm{s} \approx T_\mathrm{d}$. Therefore,

$$2\left(\frac{1-T_{\mathrm{s}}}{T_{\mathrm{s}}}\right) \gg \left(\frac{1-T_{\mathrm{ch}}}{T_{\mathrm{ch}}}\right)$$

where $T_{\mathrm{ch}} = \frac{l_{\mathrm{mfp}}}{l_{\mathrm{mfp}} + L}$ and l_{mfp}, L are the mean free path in the channel and the channel length respectively. The final condition that has to be satisfied is:

$$T_{\mathrm{s}} \ll \left(\frac{2\lambda}{2\lambda + L}\right) \quad \text{---- (1)}$$

The "T_{s}" in our paper is the WKB transmission through the source/drain Schottky barrier and is a function of the energy under consideration. The condition derived above needs to be satisfied in the energy range where most of the current flows to validate our claim that in the off-state of the device the transmission through device can be approximated to be dominated by the source/drain-to-channel transmission.

For example, at an energy *E*, in the tail of the Fermi-Dirac distribution, if the barrier defined in the channel is lower than this energy, the source-to-channel transmission becomes unity. In such a case, the scattering in the channel dominates the transmission through the device. However, this current makes up a negligible part of the total current through the device. In order to suitably describe this effect, we normalize T_{s} as follows:

$$T_{\mathrm{s,norm}} = \frac{\int_{-20k_{\mathrm{B}}T}^{20k_{\mathrm{B}}T} T_{\mathrm{wkb}} \cdot f_{\mathrm{s}}\, dE}{\int_{-20k_{\mathrm{B}}T}^{20k_{\mathrm{B}}T} f_{\mathrm{s}}\, dE} \quad \text{---- (2)}$$

where, T_{wkb} is the WKB transmission probability through a triangular Schottky barrier, f_{s} is the source Fermi-Dirac distribution and E is the energy. The limits are chosen to include energies in the V_{ds} window.

To calculate the WKB transmission probability, we assumed the parameters used for BP in our study, i.e $m^*=0.15m_0$, $\lambda=10$nm, $\Phi_{\mathrm{sb}}=0.1$ to 0.5eV. The following calculations assume a temperature of 300K.

The dashed line in the supplementary fig.1 corresponds to the limit set by equation (1.1) for l_{mfp} = 1nm and L=400nm (the channel length of our devices). Here we assumed a rather small l_{mfp} value to show that even severe scattering inside the channel does not impact our analysis. As is evident from supplementary fig.1, the transmission through the barrier is lower than the limit set by equation (1) for all barriers. It is important to crosscheck if the Schottky barriers dominate the transmission through the device, using the above equations, before applying the model to analyze the off-state.

Supplementary Note 3

Since the holes are confined in a 2D inversion layer, the mass relevant for transport is the m^* of the heavy hole band in the direction of transport (perpendicular to the <100> confinement direction), assuming that only the lowest sub-bands contribute to transport. Donetti et al. (supplementary ref [3]) show that considering a single mass does not usually explain the experimentally observed mobility values in the device ON-state. The same is likely true for the tunneling mass we are employing to describe the device off-state. Therefore, we are using an effective hole-mass value that is an average of the different mass contributions of the anisotropic heavy-hole band.

However, the exact hole mass is not critical here, since the barrier for holes in the silicon transistors considered by us is much smaller than the electron barrier. We have observed that when we evaluate "small" barriers, changing the effective mass does NOT change the tunneling probability significantly. This point is demonstrated in supplementary note 4 for black phosphorus MOSFETs.

Supplementary Note 4. Impact of the effective mass within the model

In this section, we discuss why the Schottky barrier heights extracted using our model are not sensitive to the effective mass m^* values used as an input, while in fact the total fit of the I_{ds}-V_{gs} characteristics as illustrated in supplementary fig.2 shows a clear dependence on m^*.

As discussed in the article, the different m^*-values enter the I_{ds} calculation in particular through the transmission probability (T) where T depends exponentially on m^*. The value of m^* in the armchair and zigzag directions is 0.15 and 1.18 respectively for electrons, and 0.14 and 0.89 respectively for holes. Our devices were fabricated on wide BP-flakes (>1 μm) and not on narrow ribbons. In such wide channels, the injection occurs simultaneously into all possible directions. Due to the rather large difference in m^* between the armchair and zigzag directions (factor of 6 – 8), the lower of the two masses is expected to dominate the injection process since it provides in essence a lower resistive injection path into the channel.

In addition to the impact of m^* on T, also the number of modes (M) per unit width depends on m^*. However, because M is proportional to sqrt(m^*) a very small correction for the off-state current, especially when plotted on a log scale occurs due to the m^* impact on M.

Impact of the effective mass on the extracted barrier heights:

Specifically for BP, the hole Schottky-barrier (SB) is significantly smaller than the electron SB. For the tunneling distances we are dealing with in this study, the tunneling probability from the metal into the valence band, which impacts the slope of the hole-branch, is not impacted even when the heavier hole mass is used (see supplementary fig. 2). The electron SB on the other hand is quite large and using the heavier electron mass in the WKB expression does drastically reduce the slope of the electron-tunneling branch.

The extracted barrier height, however, is not impacted (not more than the individual error bars) when the heavier mass is used for black phosphorus. To clarify this, we present the plot in supplementary fig.2.

It is evident that the lighter of the two masses fits our experimental data (open blue circles) in line with our explanation in the first paragraph above. However, independent of the choice of m^*, the same set of barrier heights were used to generate the black and red lines in supplementary fig.2. If care is taken to match the minimum current point, the Schottky barrier height extracted from our technique is rather robust to changes in the effective mass.

Supplementary References

[1] Xie, Q., Xu, J., & Taur, Y. (2012). Review and critique of analytic models of MOSFET short-channel effects in subthreshold. *IEEE Transactions on Electron Devices*, *59*(6), 1569–1579.

[2] Datta, S. Electronic transport in Mesoscopic Systems. Cambridge University Press, 1995; pp. 48-65.

[3] Donetti, L., Gámiz, F., Thomas, S., Whall, T. E., Leadley, D. R., Hellström, P.-E., Östling, M. (2011). Hole effective mass in silicon inversion layers with different substrate orientations and channel directions. *Journal of Applied Physics*, *110*(6), 063711.
